# Cephalad misplacement of a pulmonary artery catheter in a patient with a preexisting Hickman catheter

**DOI:** 10.1186/s12871-021-01254-4

**Published:** 2021-06-01

**Authors:** Hoon Choi, Joon Pyo Jeon, Jaewon Huh, Youme Kim, Wonjung Hwang

**Affiliations:** Department of Anesthesiology and Pain, Seoul St. Mary’s Hospital, College of Medicine, The Catholic University of Korea, 222, Banpo-daero, Seocho-gu, Seoul, 06591 Republic of Korea

**Keywords:** Catheterization, swan-Ganz, Anesthesia, cardiac procedures, Intraoperative complications

## Abstract

**Background:**

Pulmonary artery catheter insertion is a routine practice in high-risk patients undergoing cardiac surgery. However, pulmonary artery catheter insertion is associated with numerous complications that can be devastating to the patient. Incorrect placement is an overlooked complication with few case reports to date.

**Case presentation:**

An 18-year-old male patient underwent elective mitral valve replacement due to severe mitral valve regurgitation. The patient had a history of synovial sarcoma, and Hickman catheter had been inserted in the right internal jugular vein for systemic chemotherapy. We made multiple attempts to position the pulmonary artery catheter in the correct position but failed. A chest radiography revealed that the pulmonary artery catheter was bent and pointed in the cephalad direction. Removal of the pulmonary artery catheter was successful, and the patient was discharged 10 days after the surgery without complications.

**Conclusions:**

To prevent misplacement of the PAC, clinicians should be aware of multiple risk factors in difficult PAC placement, and be prepared to utilize adjunctive methods, such as TEE and fluoroscopy.

## Background

Pulmonary artery catheter (PAC) insertion is a routine practice in high-risk patients undergoing cardiac surgery. Although there are controversies regarding the PAC, many clinicians agree that PAC measurements may guide therapy in patients with right-sided heart failure or pulmonary hypertension [1]. PAC may help to assess therapy in the setting of severe cardiac dysfunction from valvular or ischemic etiology. Moreover, the PAC is the only modality that can acquire parameters such as continuous cardiac output and real-time pulmonary artery (PA) venous blood oxygen saturation [2, 3].

However, PAC insertion is associated with numerous complications that can be devastating to the patient. Known complications include arrhythmia [4], complete heart block [5], pulmonary infarction [6], catheter knotting and entrapment [7, 8], valvular damage [9, 10], thrombocytopenia [11, 12], thrombus formation [13], balloon rupture [2], ventricular perforation [14], and incorrect placement [15–20]. Among these, incorrect placement is an overlooked complication with few case reports to date. Here, we present a case of cephalad PAC misplacement in the right internal jugular vein (RIJV).

## Case presentation

An 18-year-old male patient underwent elective mitral valve replacement (MVR) due to severe mitral valve regurgitation (MR). The patient had a history of synovial sarcoma in the left subscapularis muscle and hypereosinophilic syndrome. Ten months before surgery, a 12 French (F) Hickman catheter (Hickman® 12F Dual-Lumen CV Catheter; Bard Access Systems, Inc., Salt Lake City, UT, USA) had been inserted into the RIJV for systemic chemotherapy. The patient had undergone mitral valvuloplasty with the same indication 5 months before the surgery. At the time, we had assumed that PAC insertion was mandatory to monitor right-side heart pressure and pulmonary artery pressure (PAP). Therefore, we evaluated the patient’s RIJV and the superior vena cava (SVC) diameter by chest computed tomography (CT), and concluded that the placement of a 9F advanced venous access (AVA) catheter (AVA High-Flow Device; Edwards Life Sciences, Irvine, CA, USA) for PAC insertion was possible. As expected, an 8F PAC (Swan-Ganz CCOmbo V; Edwards Life Sciences) was inserted and maintained until the day after surgery without any complications. The initial measured PAP after PAC placement was 37/12 mmHg.

After 5 months from the previous surgery, the patient complained of dyspnea and edema. Follow-up echocardiography showed severe MR, moderate tricuspid valve regurgitation (TR), and pulmonary hypertension with a systolic pressure of 72 mmHg. Therefore, we decided to reinsert the PAC. As before, evaluation of the patient’s vessel was performed based on the patient’s new chest CT performed a day before the surgery, and no interval change was noted. We decided to insert the PAC through the RIJV, because the RIJV provides the most direct route, and prior PAC placement through the RIJV was successful. After induction of anesthesia, the AVA catheter was inserted into the RIJV without any complications. The PAC was inserted into the PAC introducer sheath and advanced with monitoring of the pressure waveform. The right ventricle (RV) pressure waveform was obtained at a depth of 45 cm, and the RV pressure was 65/15 mmHg. However, although the PAC was inserted more than 60 cm, we could not obtain the PA waveform and only the RV waveform was seen. The balloon was deflated and withdrawn into the right atrium (RA), and two more failed attempts were made at the neutral bed position. A fourth attempt was made with a change in position, to the head-up position with right lateral tilt after entering the RV, but the PA could not be entered. We concluded that the difficult PAC placement was due to moderate TR and pulmonary hypertension, and that surgical repair may facilitate PAC placement. Therefore, we decided to proceed with the surgery and reposition the PAC after termination of cardiopulmonary bypass (CPB).

**Fig. 1:**
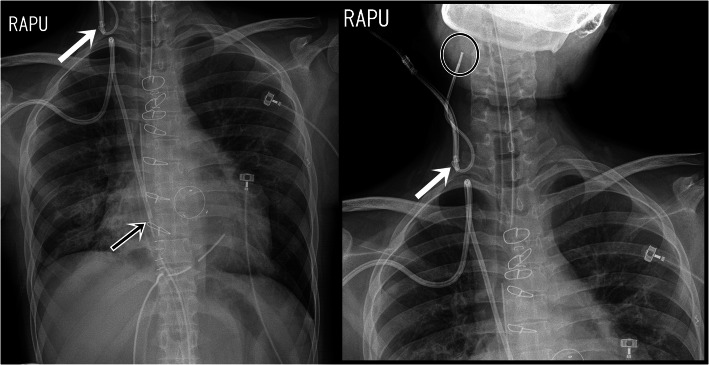
Cephalad misplacement of the pulmonary artery catheter. White arrow points to the exit of the introducer sheath, where the pulmonary artery catheter was bent and pointed in the cephalad direction in the right internal jugular vein. Black arrow points to the Hickman catheter tip in the right atrium. Black circle indicates the tip of the pulmonary artery catheter in the cranial right internal jugular vein

After successful weaning from CPB, transesophageal echocardiography (TEE) showed a well-functioning prosthetic valve and a reduction in TR, from moderate to mild. The patient was in a slight head-up position, as requested by the surgeon for visualization of the surgical field. We made another attempt to place the PAC in the PA under TEE guidance. However, it was difficult to manipulate the TEE probe and the PAC simultaneously. Moreover, visualization of the PAC with TEE was hindered by acoustic shadowing of the prosthetic mitral valve. We could not obtain the RV pressure waveform even at a depth of 50 cm, and the pressure waveform consistently showed the RA waveform. We decided to deflate the balloon and withdraw the catheter. While withdrawing the catheter, resistance was felt at 30 cm, and the catheter could not be withdrawn further. The catheter could not be visualized in the right heart chambers or the SVC with TEE, and therefore chest radiography was performed after surgery. Chest radiography indicated that the PAC was bent and pointed in the cephalad direction in the RIJV (Fig. 1). We decided to remove the AVA catheter and the PAC as one unit; removal was successful, without any resistance. The patient was discharged 10 days after surgery without complications.

## Discussion and conclusions

The PAC is favored by many cardiac anesthesiologists in high-risk cardiac surgery, but there is controversy due to complications regarding PAC insertion [2, 3]. Clinical indications for PAC monitoring are shown in Table 1 [1]. The patient presented here had severe MR and pulmonary hypertension, and there was a possibility of resultant right-sided heart failure. Therefore, PAC monitoring was considered necessary in this case.

**Table 1:** Clinical indications for pulmonary artery catheter monitoring in cardiac surgery

Right-sided heart failure, pulmonary hypertension	
Severe left-sided heart failure not responsive to therapy	
Cardiogenic or septic shock or with multiple-organ failure	
Orthotopic heart transplantation	
Left ventricular-assist device implantation	

Complications related to the PAC include arrhythmias [4], complete heart block [5], pulmonary infarction [6], catheter knotting and entrapment [7, 8], valvular damage [9, 10], thrombocytopenia [11, 12], thrombus formation [13], balloon rupture [2], ventricular perforation [14], and incorrect placement [15–20]. Complete heart block is possible in patients with preexisting LBBB due to electrical irritability from the PAC tip causing transient right bundle branch block as it passes through the right ventricular outflow tract [5]. Mild thrombocytopenia is possible, and although heparin-coated PACs may reduce this risk, these catheters can trigger heparin-induced thrombocytopenia [11, 12]. Misplacement of the PAC occurred in our patient. Spontaneous wedging of the catheter during CPB is the most frequent form of malposition [2]. Although there have been few case reports regarding PAC misplacement, abnormal sites such as the liver, coronary sinus, pulmonary vein, and right subclavian vein have been described [15, 17, 19, 20]. In addition, looping of the PAC around an inferior vena cava filter and a left ventricular assist device has been described [16, 18]. In patients with a persistent foramen ovale, or an atrial or ventricular septal defect, placement of the PAC in the left side of the heart is possible. Reports of PAC placement toward the cephalad direction are limited, but there have been reports of central venous catheters bent upward in the RIJV [21, 22]. Catheter misplacement in the cephalad direction can lead to serious complications, including thrombosis and hemorrhage [23]. Early recognition and withdrawal of the PAC in our patient led to hospital discharge without complications.

We hypothesized misplacement of the PAC due to the patient’s position during PAC insertion and the presence of another catheter in the same vein. The balloon of the PAC tends to float to nondependent regions. Therefore, the position of the patient influences the passage of the PAC. In this case, the surgeon requested a head-up position to aid visualization of the surgical field. This position may have affected the balloon of the PAC, causing it to float toward the head. In addition, the presence of a Hickman catheter in the RIJV may have served as an additional complicating factor. Although inserting two different catheters into the RIJV is known to be feasible [24], there has been a report of failed PAC insertion in the presence of two catheters in the RIJV [25]. In our case, thorough assessment of the RIJV and the SVC was performed with chest CT prior to insertion of the AVA catheter, and the insertion was successful without complications. However, the tip of the introducer sheath was placed more distal from the heart than the insertion site of the Hickman catheter, as revealed by chest radiographs. Therefore, the Hickman catheter may have interfered with passage of the PAC with the inflated balloon in this case.

Difficulty in PAC placement was anticipated due to the patient’s cardiac condition. It has been documented that enlarged cardiac chambers, low cardiac output, pulmonary hypertension, and TR are related to difficult PAC positioning [2, 3]. Our patient presented with enlarged cardiac chambers, pulmonary hypertension, and moderate TR at the time of this event. Therefore, unlike the previous surgery, successful PAC placement could not be achieved easily despite proper positioning of the patient after introducer sheath insertion. Normally, placing the patient in a head-down position aids flotation from the RA to the RV, and repositioning the patient to achieve a right lateral tilt, with the head tilted slightly upward, aids flotation from the RV to the PA [2, 3]. TEE or fluoroscopy can be used as alternatives to conventional waveform-based PAC placement with expertise hands [26–28]. Both adjunct methods have been shown efficacy in potentially difficult cases. Many cardiac anesthesiologists prefer TEE because it is a routine monitoring method in cardiac surgery. Moreover, TEE has advantages over fluoroscopy in that the latter is not always readily available and involves exposure to radiation [27, 28]. Three TEE views can aid advancement of the PAC; a midesophageal modified bicaval view when passing through the tricuspid valve; a midesophageal right ventricular inflow-outflow view when maneuvering through the RV and RV outflow tract; and a midesophageal ascending aortic short-axis view when confirming the final position of the PAC at the junction of the main PA and the right PA [27, 28]. When difficult PAC placement is anticipated, TEE is recommended along with pressure waveform analysis. However, we had used only pressure waveform-dependent PAC insertion in our most difficult cases and were not familiar with TEE-guided PAC positioning. Although TEE was available at the time of the final attempt at PAC placement in this case, we could not visualize the PAC with TEE, partially due to acoustic shadowing of the prosthetic mitral valve. As many cardiac surgery patients present with risk factors for difficult PAC placement, cardiac anesthesiologists should be experienced in the practice of placing the PAC with TEE.

This case report described PAC insertion in a patient with a preexisting Hickman catheter in the RIJV, which led to bending of the PAC and placement of the PAC in the cranial RIJV. To prevent misplacement of the PAC, clinicians should be aware of multiple risk factors in difficult PAC placement, and be prepared to utilize adjunctive methods, such as TEE and fluoroscopy.

## Data Availability

All data related to this case report are contained within the manuscript.

## Abbreviations

AVA: Advanced venous access
CPB: Cardiopulmonary bypass
CT: Computed tomography
F: French
MR: Mitral valve regurgitation
MVR: Mitral valve replacement
PA: Pulmonary artery
PAC: Pulmonary artery catheter
PAP: Pulmonary artery pressure
RA: Right atrium
RIJV: Right internal jugular vein
RV: Right ventricle
SVC: Superior vena cava
TEE: Transesophageal echocardiography
TR: Tricuspid valve regurgitation
